# Scientific Items

**Published:** 1883-02-20

**Authors:** 


### SCIENTIFIC ITEMS.

**Burdensome Inventions.**—In any estimate of the public value of several recent inventions which have been received with great apparent favor, the important fact must not be lost sight of that these inventions are a burdensome tax upon the people, for which, in a majority of cases, no corresponding benefits are received.

The introduction of the telephone, or its use to a very large extent, is, in a true sense, *compulsory*. It is not introduced into the counting-rooms, stores, and dwellings because it is needed or wanted, but because pride or competition influences the patron. In many instances where it has been introduced into dwellings, pride and curiosity combined have led to the act—not any pressing want or necessity. It is learned by Mr. A. that his neighbor Mr. B., a wealthy man, perhaps, has had the "wires" brought to his house, and all the family are shouting to butchers and grocers through the little round hole in the polished box on the wall. Mr. A. and his good wife decide that they must have all the "facilities" for instantaneous communication that Mr. B. has, although their pecuniary resources are barely sufficient for the absolute needs of the family, and so the telephone man is sent for, and forthwith the A. family are shouting through the hole in the box as loudly and frequently as the B. family. This "great convenience" adds fifty dollars a year to the household expenses, a sum which cannot well be afforded.—*Journal of Chemistry.*

**Progress of Electrical Lighting.**—There is at the present time great activity among the rival electric lighting companies to bring to notice their various devices. To ordinary observation it would appear that the new method of lighting had passed the stage of experiment, and becomes firmly established as the successful rival of gas. This is not true, however, and it is a matter of doubt if it ever becomes true. A vast number of formidable difficulties have been overcome which stood in the way of practical success, but there remains others which are of no mean importance. The time is near when decisive action will be taken to prevent the placing of conducting wires above ground, and it appears at present as if the difficulties in the way of the success of buried wires were insurmountable. The enormous expense of the undertaking and the difficulties of insulation are among the obstacles of the greatest moment. Mr. Edison, in New York, has met with only partial success, and at the present time appears to be inactive. The cost of putting down his street conductors was excessive, and the true cost will probably not be known outside of interested parties.—*Journal of Chemistry.*

**The Signal Service** office estimates that ships containing at least $13,000,000 of property, besides many lives, were saved from running into the disastrous cyclone in October by the warning it gave. The money thus saved in this one storm would pay the expense of the Service for ten years.—*Mechanical News.*

**Electricity in the Parlor, in the Kitchen, and Everywhere.**—A writer in "The Electrician," a monthly journal published in New York, says: 1. "Shall we ever have the electric light in our houses? 2. Shall we have to produce the electric current ourselves? 3. Will it be cheaper than gas or even as cheap? All these questions he answers affirmatively. He says: "There is not the slightest doubt in my mind that we shall have the light in our houses; and on the other hand it will not be necessary that the consumer should supply himself with the electric current any more than at the present time he is obliged to supply himself with gas. It would, however, be just as possible for him to do so." The third question, he says, requires much consideration, but he has no doubt that electric lighting will ultimately be cheaper than gas.—*Scientific Exchange.*

**The** Boston Journal of Chemistry thinks that the headaches that many thousands wake up with every morning are brought about by kerosene lamps "turned down low." A small flame in a lamp chimney does not cause enough draft to insure complete combustion, and slumberers breathe carbon and carbonic acid gas as literally as if they stood over the chimney of a petroleum refinery. A little light may be supplied in a bed-chamber, if any is required, by a specially prepared taper, by a candle, or by a wick floated in animal or vegetable oil; but the "turned-down" kerosene lamp cannot be used except to one's disadvantage.—*Ex.*

**Signaling by Electricity.**—A method of signaling by means of electric balloons was tried recently in Paris by MM. Mangin and Baudet. The balloon made of paper rendered translucent was about eight feet in diameter and was filled with pure hydrogen. A Swan lamp was fitted inside and a light rope, carrying two copper wires, was attached. When the circuit was completed the whole balloon appeared to be a globe of fire. By switching the current off and on, the Morse code can be spelled out, and thus electric balloons of this kind can be used for signaling purposes.—*The Electrician.*

**A New** branch of industry has sprung up in Sweden lately—the fabrication of paper from moss—not from the living plant, but from the bleached and blanched remains of mosses that lived centuries ago, and of which enormous masses have accumulated in most parts of Sweden. A manufactory of paper from this material has begun operations near Joenkaeping, and is turning out paper in all degrees of excellence, from tissue to sheets three-quarters of an inch in thickness. These latter are harder than wood.—*Ibid.*

**To Make Paper** which shall be luminous in the dark, it is sufficient to mingle with the pulp the following ingredients in their proportions: Water, 10 parts; pulp, 40 parts; phosphorescent powder, 10 parts; gelatine, 1 part; bichromate of potash, 1 part. The paper will also be waterproof.—*Ex.*

